# Molecular modeling, dynamics simulations, and binding efficiency of berberine derivatives: A new group of RAF inhibitors for cancer treatment

**DOI:** 10.1371/journal.pone.0193941

**Published:** 2018-03-22

**Authors:** Parham Jabbarzadeh Kaboli, Patimah Ismail, King-Hwa Ling

**Affiliations:** 1 Department of Biomedical Science, Faculty of Medicine and Health Sciences, Universiti Putra Malaysia, Serdang, Selangor, Malaysia; 2 Genetics and Regenerative Medicine Research Centre, Faculty of Medicine and Health Sciences, Universiti Putra Malaysia, Serdang, Selangor, Malaysia; University of Crete, GREECE

## Abstract

RAF kinases are a family of enzymes in the MAP kinase pathway that contribute to the development of different types of cancer. BRAF is the most important member of RAF kinases. BRAF mutations have been detected in 7% of all cancers and 66% of melanomas; as such, the FDA has approved a few BRAF inhibitor drugs to date. However, BRAF can activate CRAF leading to resistance to BRAF inhibitors. Berberine (BBR) is an alkaloid that is widely distributed in different plant species. Several studies have been carried out on the anti-cancer effects of BBR but direct targets of BBR are unknown. In this study, interactions of BBR derivatives against BRAF and CRAF kinases were modeled and predicted using an *in silico*-based approach. To analyze and identify the residues important in BRAF docking, we modeled interactions of ATP, the universal substrate of BRAF, and found that Lys483 and Asp594 are the most important residues involved in both ATP and BBR binding [(The average score = -11.5 kcal/mol (ATP); Range of scores = -7.78 to -9.55 kcal/mol (BBR)]. In addition to these polar residues, Trp530 and Phe583 are also applicable to the molecular docking of BRAF. We also observed that Asp593 was excluded from the enzyme cavity, while Phe594 was included inside the cavity, making the enzyme inactive. Finally, three alternatives for BBR were identified with dual RAF inhibition effects [The best scores against BRAF = -11.62 kcal/mol (BBR-7), -10.64 kcal/mol (BBR-9), and -11.01 kcal/mol (BBR-10); the best scores against CRAF = -9.68 kcal/mol (BBR-7), -9.60 kcal/mol (BBR-9), and -9.20 kcal/mol (BBR-10)]. Direct effects of BBR derivatives against BRAF and CRAF kinases had not yet been reported previously, and, thus, for the first time, we report three cycloprotoberberines as lead compounds against RAF kinases.

## Introduction

Several studies have evaluated the anti-cancer effects of berberine (BBR), a plant-based alkaloid, on several signaling targets [1,2]. A membranous protein inhibited by BBR, EGF (epidermal growth factor) receptor, is a crucial biomarker of cancer [3]. BBR has been shown to downregulate EGF receptor gene expression [4]. Meanwhile, BBR can also inhibit the expression of CD147, another integral membrane protein that interacts with cytoplasmic signaling molecules [5]. PI3K/Akt (phosphoinositide 3-kinase/Akt) and MAP pathway (RAF/MEK/ERK) are two crucial cell signaling pathways and play significant roles in regulating gene expression and cell proliferation [6]. Both pathways are involved in complex cytoplasmic signaling networks, and are linked to membranous receptors such as EGF receptor and Her2, as well as nuclear transcription factors [1]. Several studies showed that BBR inhibits Ras, Raf, MEK, and PI3K activities [7]. Down-regulation of BRAF promotes berberine-induced apoptosis and results in the inhibition of the MAPK pathway [8].

RAF kinases are a family of enzymes containing serine/threonine OH groups [9]. There are three isoforms of RAF as follows: A-RAF, BRAF, and CRAF [10]. MAP (mitogen-activated protein) kinase signaling pathway initiation by Ras activation leads to the phosphorylation of RAF kinases. Ras-activated RAF kinases then activate another kinase called MEK, resulting in a kinase cascade toward the nucleus, which in turn promotes cell proliferation and invasiveness in cancers [11,12].

Melanoma is a type of skin cancer caused by the abnormal formation of melanocytes. The BRAF oncoprotein, discovered in 1988, is associated with nearly 66% of melanomas and 12% of colorectal cancers [13]. BRAF is a major target of therapies, as it is the most frequently mutated protein kinase in human cancers [14]. Moreover, in nearly 7% of all cancers, mutations in the BRAF gene leads to MAP kinase pathway over-activation [15]. The most common BRAF mutation, out of more than 30 BRAF mutations [16], is V600E [17]. The BRAF^V600E^ mutation results in 500-fold greater constitutive kinase activity compared to the BRAF wild type, and several BRAF^V600E^ inhibitors have been designed [4, 7].

Sorafenib is a multi-kinase inhibitor in which BRAF serine/threonine kinase and EGFR tyrosine receptor kinase are inhibited. Sorafenib is approved by US FDA for the treatment of some types of cancer, such as advanced renal cell carcinoma, hepatocellular carcinoma, and radioactive iodine-resistant advanced thyroid carcinoma [18]. In 2011, vemurafenib (Zelboraf^®^), a more specific BRAF inhibitor, was approved by the FDA for metastatic melanoma and is under evaluation for colorectal and thyroid cancers [19]. Since nearly 50% of BRAF mutations have the V600E mutation, vemurafenib is a crucial anti-melanoma and anti-metastatic drug due to its specific inhibition of BRAF^V600E^ [20].

Treatment with BRAF inhibitors may result in the development of drug resistance which limits their utility [12]. Metastatic melanoma may cause resistance mechanisms, helping these cancer cells evade the immune system. Genetic mutations may also accumulate, which can activate alternative signaling pathways like the PI3K/AKT/mTOR pathway [10]. Most patients administered vemurafenib ultimately develop resistance against it. In case of BRAF inhibition, CRAF promotes alternative signaling pathways such as PI3K/Akt/mTOR pathway [21,22]. Therefore, identifying other BRAF inhibitors that act as dual inhibitors of BRAF/CRAF is of key importance for cancer research [23].

In this study, compounds structurally similar to berberine were evaluated to predict the most effective compounds. The aim of this study was to identify alternative BRAF inhibitors for sorafenib and vemurafenib with the same therapeutic efficacy and properties with less toxicity. The objectives of this study included the following: (1) to identify how BBR and its relatives target BRAF kinase, (2) to identify optimal berberine analogues by which CRAF can be strongly inhibited, and (3) to validate optimal BBR derivatives by molecular dynamics simulations.

## Methods

### Ligand selection and molecular docking

Using PubChem database, we virtually screened all compounds similar to berberine (PubChem CID: 2353). We found 1,544 compounds, which were sorted based on chemical properties, such as molecular weight, H-bond donor, H-bond acceptor, formal charge, and total formal charge (**Table 1**).

**Table 1:** Physico-chemical properties of selected ligands.

Drug-like	PubChem CID	MW (Da)	nHBA	nHBD	nTDOF	nTFC	nRot	nRB	LogP	TPSA (Å2)
BBR	2353	336.36	4	0	2	1	2	27	2.473	39.93
BBR-2	3005463	406.17	5	0	6	1	6	28	3.734	57.87
BBR-3	49865302	392.15	5	0	5	1	5	28	3.165	57.87
BBR-4	72704	322.11	4	1	2	1	1	27	2.152	50.93
BBR-5	3084288	322.11	4	1	2	1	1	27	2.152	50.93
BBR-6	15061301	364.12	5	0	3	1	3	28	2.343	57.87
BBR-7	-	481.15	4	0	5	1	5	35	3.65	40.16
BBR-8	10790783	350.14	4	0	3	1	3	27	2.896	39.93
BBR-9	-	479.13	4	0	5	1	5	35	4.5	40.16
BBR-10	-	495.17	4	0	5	1	5	36	4.73	40.16
Vemurafenib	42611257	489.07	6	2	7	0	7	29	2.786	96.01
Sorafenib	216239	464.09	7	3	6	0	9	25	1.568	91.82

**Fig 1:**
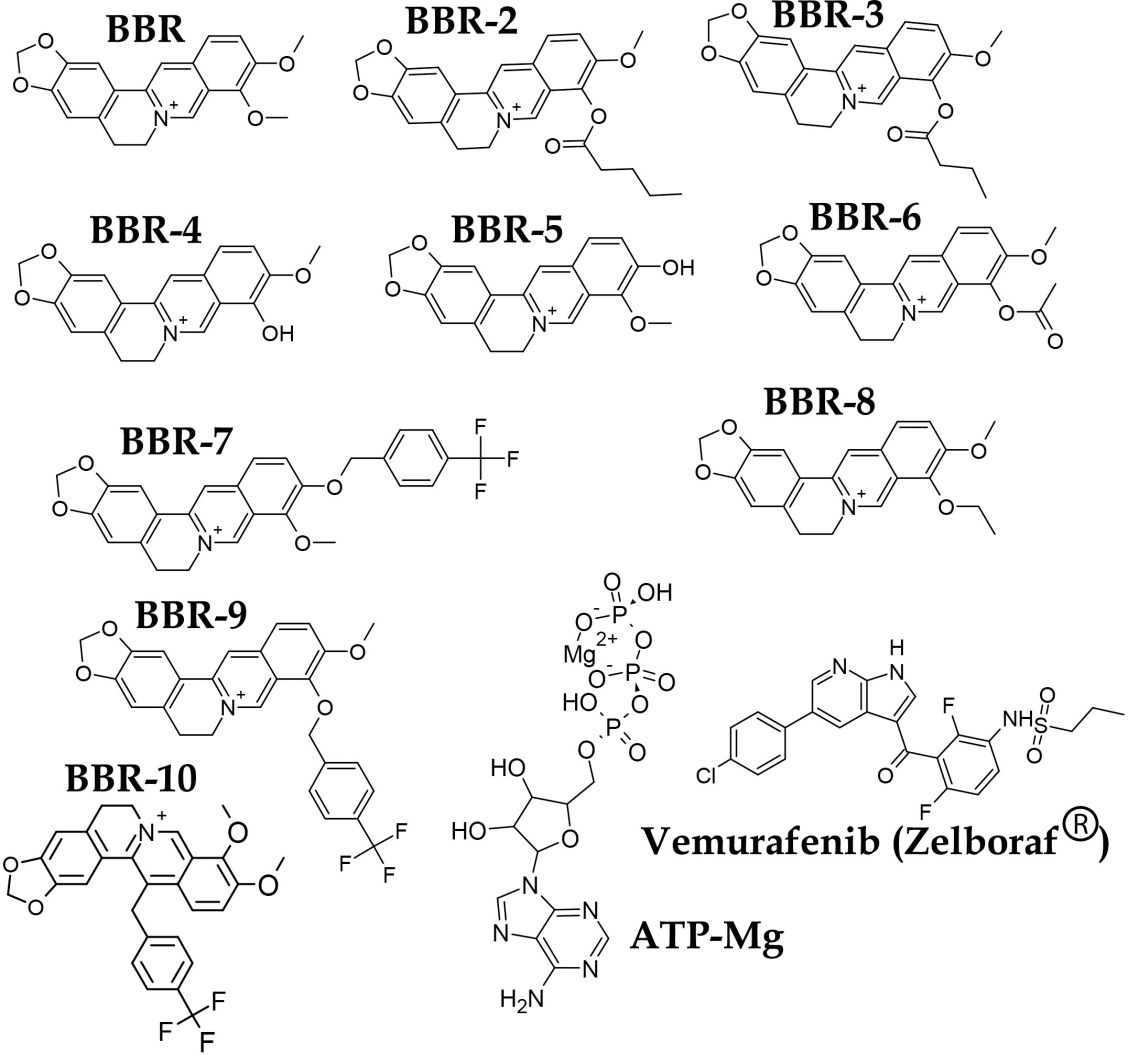
Chemical structures of optimal berberine derivatives and controls. Ball and Stick models show the optimized structures for molecular docking.

Lipinski’s rule of five was verified for 1,544 derivatives, among which 485 ligands passed the test [24]. These 485 compounds were chosen and their SDF files were downloaded and prepared for docking. In addition to BBR derivatives, vemurafenib and ATP-Mg were used as controls in the present study. ACD/ChemSketch 12.01 was used for *in silico* ligand generation, and pdb-formatted files were prepared for all compounds. To prepare and optimize the ligands for docking, polar hydrogen atoms were inserted, torsional degrees of freedom (nTDOF) were determined, and Gasteiger charges were calculated for all generated ligands. Final suitable berberine derivatives and controls used are depicted in **Fig 1**. The top 10 ligands ranked based on thermodynamics parameters were shown here.

Five crystal structures were downloaded for the BRAF kinase domain (PDB IDs: 1uwj, 1uwh, 3c4c, 3og7, and 3psd) from the RCSB protein data bank, and all generated ligands were docked to all BRAF crystals; however, a single CRAF kinase domain was found on the RCSB protein data bank (PDB ID: 3omv) [25]. Water molecules and original ligands were removed from the downloaded pdb files (**Table 2**), and the crystals were optimized and energetically minimized by Swiss-PdbViewer 4.1.0. Optimized crystals were used only for docking the generated ligands.

**Table 2:** Crystal structures obtained from the RSCB protein data bank.

Protein	Pdb ID	Conformation	Phenotype	Resolution (Å)	R-value work	R-value free
BRAF	1uwh [27]	DFG-ASP out ^1^	Wild-type	2.95	0.222	0.257
	1uwj [27]	DFG-ASP out	Mutated	3.5	0.275	0.357
	3c4c [28]	DFG-ASP in ^2^	Mutated	2.57	0.259	0.303
	3og7 [14]	DFG-ASP in	Mutated	2.45	0.213	0.257
	3psd [29]	DFG-ASP in	Wild-type	3.6	0.277	0.335
CRAF	3omv [25]	DFG-ASP in	Wild-type	4.0	0.280	0.364

DFG: D = Aspartate, F = Phenylalanine, G = Glycine

^1^Inactive kinase

^2^Active kinase.

AutoDock 4.2 was used to dock all ligands [26] (the grid and docking configuration set ups are available in the online edition of this article). To set up the grid box, points were adjusted to 60 in each dimension and optimized coordinates of the CA atoms of Lys482 (PDB IDs: 1uwh and 1uwj), Lys483 (PDB IDs: 3c4c, 3og7, and 3psd), and Lys375 (PDB ID: 3omv) were determined as the center of the grid box in the corresponding docking.

### Ligand topology and molecular dynamics simulations

Hydrogen atoms were added to ligands evaluated for molecular dynamics simulation. We carried out semi-empirical quantum mechanical calculations (QM) for ligands with atom numbers of more than 40 (BBR, BBR-7, BBR-9, BBR-10, sorafenib, and vemurafenib) to create ligand topology files as input files required for molecular dynamics simulations. Bonded parameters, such as bond length, bond angles, and dihedral angles, as well as the initial partial atomic charges, were calculated for the united-atom version of the 53a6 GROMOS force field. Accordingly, the parameters and topology files were generated and optimized using the Automated Force Field Topology Builder (ATB) version 2.2 [30,31].

All molecular dynamics (MD) simulations were carried out using GROMACS v.5.0.4 under the Ubuntu Linux platform version 15.0.4 with a GROMOS 53a6 force field [32]. Water systems were generated such that each docked protein was placed in the center of a triclinic box with a minimum distance between the solutes and the edge of the box of 1.0nm. Accordingly, all solutes were solvated using the SPC water model [32]. Adding sodium and chloride ions neutralized the systems. All configurations used were based on previously published protocols [2,33,34].

### Molecular visualization

To demonstrate inter-molecular interactions (e.g., hydrophobic, h-bonds, halogen bonds, and π/aromatic interactions), the Accelrys Discovery Studio Visualizer version 4.1 (DS) was applied. In addition to DS, intermolecular hydrogen-bonds were also checked using the LigPlot^+^ v.1.4.5, PyMol v.1.7.4.4, UCSF Chimera.1.10.2, and AutoDock 4.2 [26]. Using the UCSF Chimera and DS, we added all hydrogen bonds and any editing required for ligand topology generations. Molecular dynamics trajectories were visualized and plotted using Grace 5.0.0.

## Results

### Molecular docking of BBR derivatives on BRAF

To analyze the effects of BBR derivatives on BRAF, we selected five pdb-formatted crystal structures. Each PDB structure was built under slightly different X-ray crystallography conditions. Therefore, we repeated, compared, and calculated the means of binding energies and inhibition constants in five independent *in silico* experiments (**Table 3**).

**Table 3:** Docking information for the optimal berberine derivatives docked to kinase domains of BRAF. Run: 10; crystal = 5; iterations = 50; T = 298.15 K.

Ligand	Pdb ID	Clusters	Torsional free E (kcal mol^-1^)	Lowest E (kcal mol^-1^)	Mean E (kcal mol^-1^)	Lowest Ki (nM)	Mean Ki (nM)	pKi (M)
BBR	1uwh	2	+0.60	-8.41	-8.33	684.74		6.08
	1uwj	1	+0.60	-9.55	-9.53	100.10		
	3c4c	2	+0.60	-8.44	-8.40	647.30	832	
	3og7	1	+0.60	-7.78	-7.76	1980		
	3psd	1	+0.60	-8.60	-8.59	497.24		
BBR-2	1uwh	2	+1.79	-8.98	-8.67	262.81		6.48
	1uwj	3	+1.79	-10.38	-9.29	24.36		
	3c4c	2	+1.79	-9.44	-8.93	119.66	331	
	3og7	1	+1.79	-8.71	-8.57	412.95		
	3psd	1	+1.79	-9.81	-9.61	64.85		
BBR-3	1uwh	3	+1.49	-8.43	-8.10	658.29		
	1uwj	2	+1.49	-10.61	9.77	16.71		
	3c4c	4	+1.49	-9.14	-8.44	198.67	1258	5.90
	3og7	4	+1.49	-7.80	-7.40	1920		
	3psd	1	+1.49	-9.46	-9.33	116.92		
BBR-4	1uwh	2	+0.60	-8.05	-7.97	1260		
	1uwj	1	+0.60	-8.97	-8.96	264.39		
	3c4c	2	+0.60	-7.92	-7.82	1570	1549	5.81
	3og7	1	+0.60	-7.47	-7.45	3370		
	3psd	1	+0.60	-8.59	-8.57	507.28		
BBR-5	1uwh	1	+0.60	-8.71	-8.67	412.15		
	1uwj	1	+0.60	-9.27	-9.25	159.96		
	3c4c	2	+0.60	-7.91	-7.90	1580	776	6.11
	3og7	1	+0.60	-7.96	-7.78	1460		
	3psd	1	+0.60	-8.60	-8.80	352.40		
BBR-6	1uwh	2	+0.89	-9.22	-9.04	175.09		
	1uwj	1	+0.89	-10.89	-10.13	36.40		
	3c4c	2	+0.89	-8.95	-8.72	274.99	380	6.42
	3og7	1	+0.89	-8.21	-8.21	855.04		
	3psd	2	+0.89	-9.24	-9.20	167.57		
BBR-7	1uwh	3	+1.49	-9.66	-8.81	82.32		
	1uwj	2	+1.49	-11.44	-10.71	4.14		
	3c4c	2	+1.49	-11.62	-11.16	3.03	246	6.60
	3og7	5	+1.49	-10.46	-9.57	21.48		
	3psd	6	+1.49	-9.24	-8.5	168.34		
BBR-8	1uwh	2	+0.89	-8.93	-8.68	283.94		
	1uwj	1	+0.89	-9.94	-9.92	51.72		
	3c4c	1	+0.89	-8.78	-8.76	368.26	617	6.21
	3og7	2	+0.89	-7.94	-7.85	1520		
	3psd	2	+0.89	-8.93	-8.84	284.65		
BBR-9	1uwh	7	+1.49	-10.14	-9.26	37.12		
	1uwj	2	+1.49	-10.64	-10.56	15.85		
	3c4c	6	+1.49	-9.96	-9.62	50.32	155	6.81
	3og7	3	+1.49	-9.53	-9.41	103.08		
	3psd	3	+1.49	-10.11	-9.97	38.97		
BBR-10	1uwh	3	+1.49	-9.20	-8.65	181.31		
	1uwj	2	+1.49	-11.01	-10.64	8.47		
	3c4c	3	+1.49	-9.54	-9.22	101.76	269	6.57
	3og7	3	+1.49	-8.34	-8.35	365.89		
	3psd	2	+1.49	-9.87	-9.52	58.63		

Three active BRAFs (PDB IDs: 3og7, 3psd, and 3c4c) and two inactive BRAFs (PDB IDs: 1uwh and 1uwj) were used to compare the effects of each ligand on different BRAF conformations. We also focused on active BRAFs and, specifically BRAF^V600E^, the well-known BRAF mutation (PDB ID: 3og7).

In **Table 3**, docking information is described. The number of conformational clusters was dependent upon the size of the ligands, the number of torsional degrees of freedom, n_TDOF_, and the conformation of the active site of the enzyme (Table 1). Each ligand requires free positive torsional energy to fit suitably with the BRAF active site. For molecules with higher n_TDOF_, torsional free energy is greater than the compounds with the lower n_TDOF_, which leads to diversity in ligand conformations entering the active site in separate iterations. BBR, BBR-4, and BBR-5 have the lowest torsional free energy (torsional ΔG = +0.60 kcal/mol), while BBR-2 has the highest torsional free energy (torsional ΔG = +1.79 kcal/mol).

Estimated free energy of binding is the sum of the following energies:

1. Final Intermolecular Energy
  (vdW + Hbond + desolvation Energy + Electrostatic Energy)
2. Torsional Free Energy

For the most potent BBR derivatives, the estimated free energy should be less than -9.00 kcal/mol. As torsional free energy is a positive parameter, it affects the total binding energy that should normally be at least possible or negative. To identify the optimal BRAF inhibitors, inhibition constants (Ki) were served in the form of pKi. pKi equals–LogKi, in which the unit of Ki (nM) is converted to molar (M). After calculating the mean Ki and pKi, the optimal BRAF inhibitors were chosen based on the highest pKi. BBR and most of its derivatives are effective in targeting BRAF, with pKis greater than 6; however, we generated three novel BBR derivatives, which were not reported previously in the PubChem database.

After comparing the results of docking and molecular properties, we added a trifluoromethyl benzyl group to BBR at three positions (9, 10, and 13 for BBR-9, BBR-7, and BBR-10, respectively) [35]. This group has two features:

1. Hydrophobicity is increased.
2. Halogen bonds were generated; the chance of interacting with lysine is increased.

After performing molecular docking algorithms for these new compounds, we found that pKi of BBR-9, BBR-7, and BBR-10 were increased to 6.81, 6.60, and 6.57, respectively, whereas the pKi for BBR was 6.08. Therefore, these three novel compounds are novel BRAF inhibitors and, as such, their results were compared to the molecular docking of vemurafenib and sorafenib as positive controls.

### Molecular interactions

**Fig 2:**
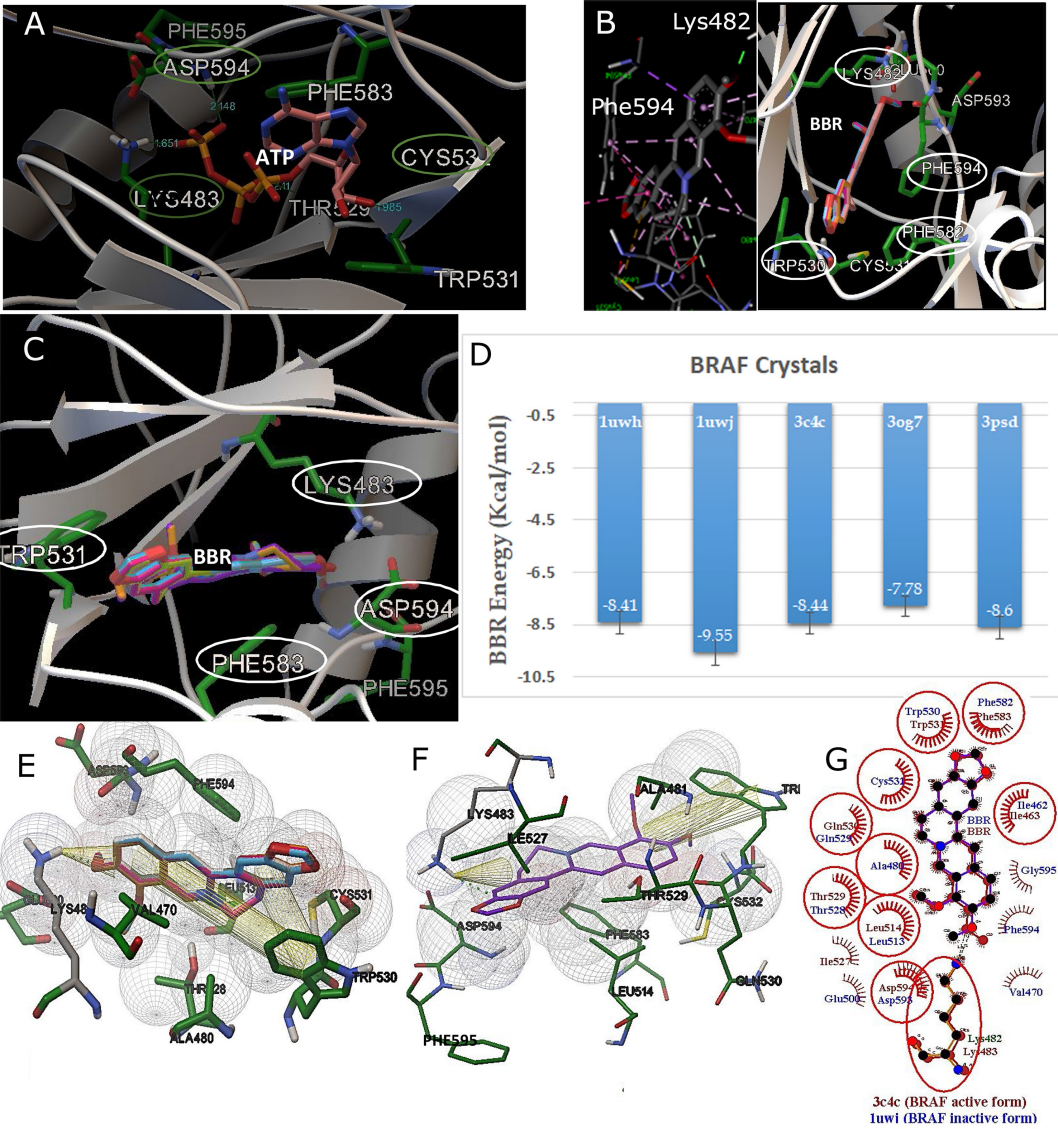
BBR docked to active and inactive BRAFs. **(A)** ATP as a normal BRAF substrate docked as a control to indicate the amino acids involved; **(B)** BBR docked to inactive BRAF to indicate lower binding energy; **(C)** BBR docked to active BRAF; **(D)** A comparison of the average binding energies of BBR in different BRAF crystals; **(E)** BBR docked to inactive BRAF; **(F)** BBR docked to active BRAF; **(G)** Comparison of BBR docked to active (3c4c) and inactive (1uwj) BRAFs. LYS plays a key role in both forms of BRAF. Lys482 (1uwj) and Lys483 (3c4c) indicate H-bond and cation-π interactions; however, in 3c4c (BRAF active form), Phe594 is out of the active site. TRP531 and PHE583 are the major amino acids involved in the π-π hydrophobic interactions (π-π and cation-π interactions). ASP594 and PHE595 are located in and out of the active site, respectively, to show the active form. The results predicts that BBR binds to both active and inactive forms of BRAF.

To identify the residues important to BRAF docking, we modeled the interactions of ATP, the universal substrate of BRAF (**Fig 2**). ATP binds to Lys483 and Asp594 [9]. In addition to these polar residues, Trp530 and Phe583 are other residues applicable to the molecular docking of BRAF. In our comparison between active and inactive forms, Asp593 was found to be located outside of the enzyme cavity and Phe594 was found to be inside the cavity, making the enzyme inactive (PDB ID: 1uwj).

The active site of the enzyme was chosen (selective approach) using the preliminary docking of ATP-Mg and molecular structure of the kinase domain. BBR-9 was predicted as a BRAF inhibitor, which is bound strongly to the kinase pocket. Residues Lys483, Phe583, Trp531, and Asp594 were recognized as the crucial residues located at the kinase domain of BRAF.

To validate whether BBR-9 is a selective BRAF-kinase inhibitor or non-selective, blind docking (randomized approach) was also performed. The minimum inhibition constants belonged to the conformations randomly docked to the kinase cavity to the residues involved in the selective approach.

In **Fig 2**, BBR binding to both forms of BRAF were compared and show the similarity of BBR interactions in both BRAF conformations. BBR was bound to both active and inactive forms with similar residue involvement. In almost all iterations, Lys483 (or Lys482 of an inactive form) was the most important residue involved in ATP-BRAF and BBR-BRAF interactions, and lysine, with its cation group (^+^NH3), is involved in H-bond and cation-π interactions. Aromatic groups of BBR play a major role in hydrophobic interactions. Two aromatic residues, Trp531 (or Trp530 of inactive BRAF) and Phe583 (or Phe582 of inactive BRAF), increased binding stability by providing π-π interactions with BBR aromatic rings.

**Fig 3:**
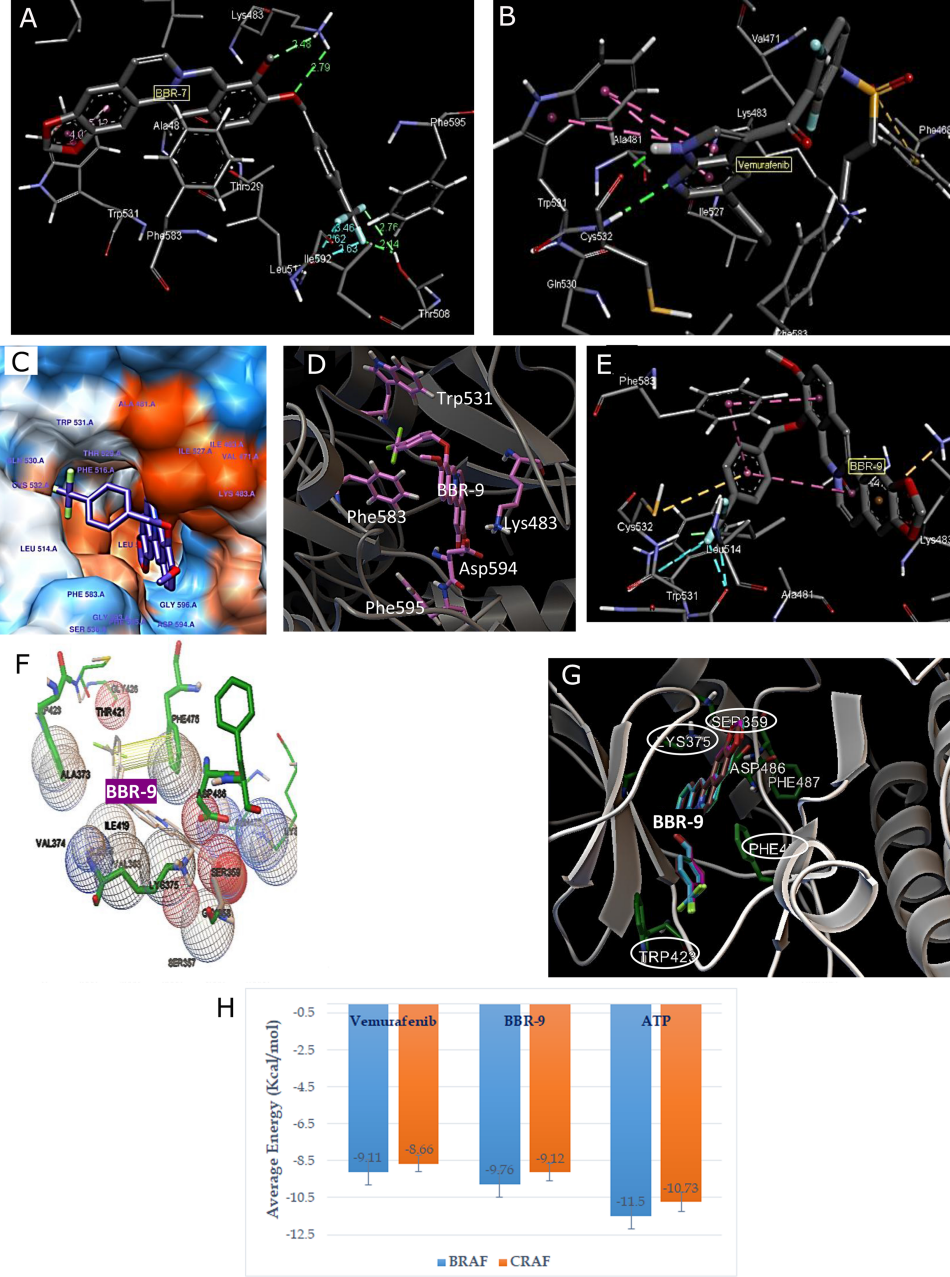
Molecular docking of BBD-7, BBR-9, and vemurafenib against BRAF and CRAF. **(A)** BBR-7/BRAF interactions; **(B)** Vemurafenib/BRAF interactions. Lys483 formed H-bonds (green lines) and Phe583 and Trp531 formed π-π interactions (purple lines); **(C, D, E)** Interactions of BBR-9 and neighboring residues of BRAF in binding cavity. Purple lines show T-shaped π-π interactions. BBR-9 indicates inter- and intra-molecular π-π interactions; **(F,G)** CRAF/BBR-9 docking; **(F)** Neighboring residues in the CRAF binding cavity; **(G)** Results from docking showing the different residues involved in CRAF/BBR-9 interactions. Lys375, Asp486, Phe475, Ser359, and Trp423 are the most important residues in CRAF docking; **(H)** The average energies of docking of BBR-9 and two controls, vemurafenib and ATP.

While face-to-face π-π interactions (π-stacking) are the most common form of π-interactions, T-shaped π-π interactions produced edge-to-face arrangement of two aromatic rings. In **Fig 3A**, BBR-7 sandwiches between Trp531 and Phe583. T-shaped π-π interaction was also seen between BBR-9 and Phe583 (**Fig 3C–3E**). The ΔGᵒ of hydrogen bonding and π-interactions can be in the range -3 to -5 kcal/mol and -1 to -5 kcal/mol, respectively.

The three novel compounds generated had the lowest binding energies and inhibition constants and the highest pKi (**Table 3**). Binding of BBR-7, BBR-9, and vemurafenib (**Fig 3**) to the continuously active form of BRAF^V600E^ (PDB ID: 3og7) was compared. We concluded that Lys483 is the most important residue, while Trp531 and Phe583 form π-π interactions.

We added additional aromatic group to different positions of BBR to generate BBR-7, BBR-9, and BBR10; therefore, the binding energies were considerably reduced, as Phe583 and Trp531 formed additional π-interactions. Thus, the additional intermolecular π-π interaction makes these novel compounds better inhibitors than BBR. In addition to the intermolecular π-interaction, BBR-9 formed intramolecular π-π interactions (**Fig 3E**), which renders it the best-fitted compound with a more rigid docking structure compared with the other BBR derivatives.

### Molecular docking of BBR-9 on CRAF

To find dual RAF inhibitors, the optimal BRAF inhibitors were selected based on pKi values and molecular docking analysis was performed for CRAF crystal (PDB ID: 3omv) with 50 iterations. Vemurafenib and ATP-Mg were also used as *in silico* positive controls for both BRAF and CRAF kinases. As pKi is a target-specific parameter, we observed that BBR-9 hds the highest pKi of 6.58; pKi was calculated based on the mean Ki of 50 iterations (**Table 4**). **Table 4** shows the comparative docking results of BRAF and CRAF. The structure of CRAF binding cavity is nearly the same as BRAF with Lys, Asp, and Phe. In **Fig 3F and 3G**, BBR-9 shows interactions with Lys375 and Asp486 of CRAF, and shows that Phe475 forms a face-to-face π-π interaction with the bended aromatic ring of BBR-9.

**Table 4:** Comparative docking information for the optimal berberine derivatives docked to BRAF and CRAF.

Target	Ligand	N	Lowest energy	Mean energy	Ki (SE^1^)	Ki (95% CI^2^)			pKi
						Lower limit (nM)	Upper limit (nM)		
CRAF	BBR	50	-7.88	-7.80	1680 (47)	1588	1772		5.77
	BBR-6	50	-8.60	-8.22	1019 (57)	907	1131		5.99
	BBR-7	50	-9.68	-8.05	1914 (48)	1820	2008		5.72
	BBR-9	50	-9.60	-9.12	265 (50)	167	363		6.58
	BBR-10	50	-9.20	-8.70	374 (43)	290	458		6.46
	Vemurafenib	50	-11.12	-8.66	975 (218)	548	1402		6.02
	ATP-MG^3^	50	-11.06	-10.73	-[26 (14)]	-[1]	-[53]		-[8.59]
BRAF	BBR	50	-9.55	-8.52	832 (326)	193	1471		6.08
	BBR-2	50	-10.38	-9.01	332 (81)	173	491		6.48
	BBR-6	50	-10.89	-9.06	378 (163)	59	698		6.42
	BBR-7	50	-11.62	-9.75	245 (110)	29	461		6.60
	BBR-9	50	-10.64	-9.76	155 (29)	98	212		6.81
	BBR-10	50	-11.01	-9.27	269 (105)	63	332		6.57
	Sorafenib	20	-9.36	-8.66	611 (36)	540	682		6.21
	Vemurafenib	50	-11.88	-9.11	550 (118)	432	668		6.26
	ATP-MG	50	-12.56	-11.50	-[8.00 (3.7)]	-[0.8]	-[15.2]		-[9.10]

^1^Standard error of the mean

^2^Confidence interval

^3^As ATP is the substrate of kinase enzymes, dissociation constants (Kd) are shown in brackets.

Based on **Table 4,** we predicted that BBR-9 and BBR-10 may be candidates as dual RAF inhibitors stronger than BBR. We then validated the stability of docking using MD analysis.

### Computational validation by MD analysis

**Fig 4:**
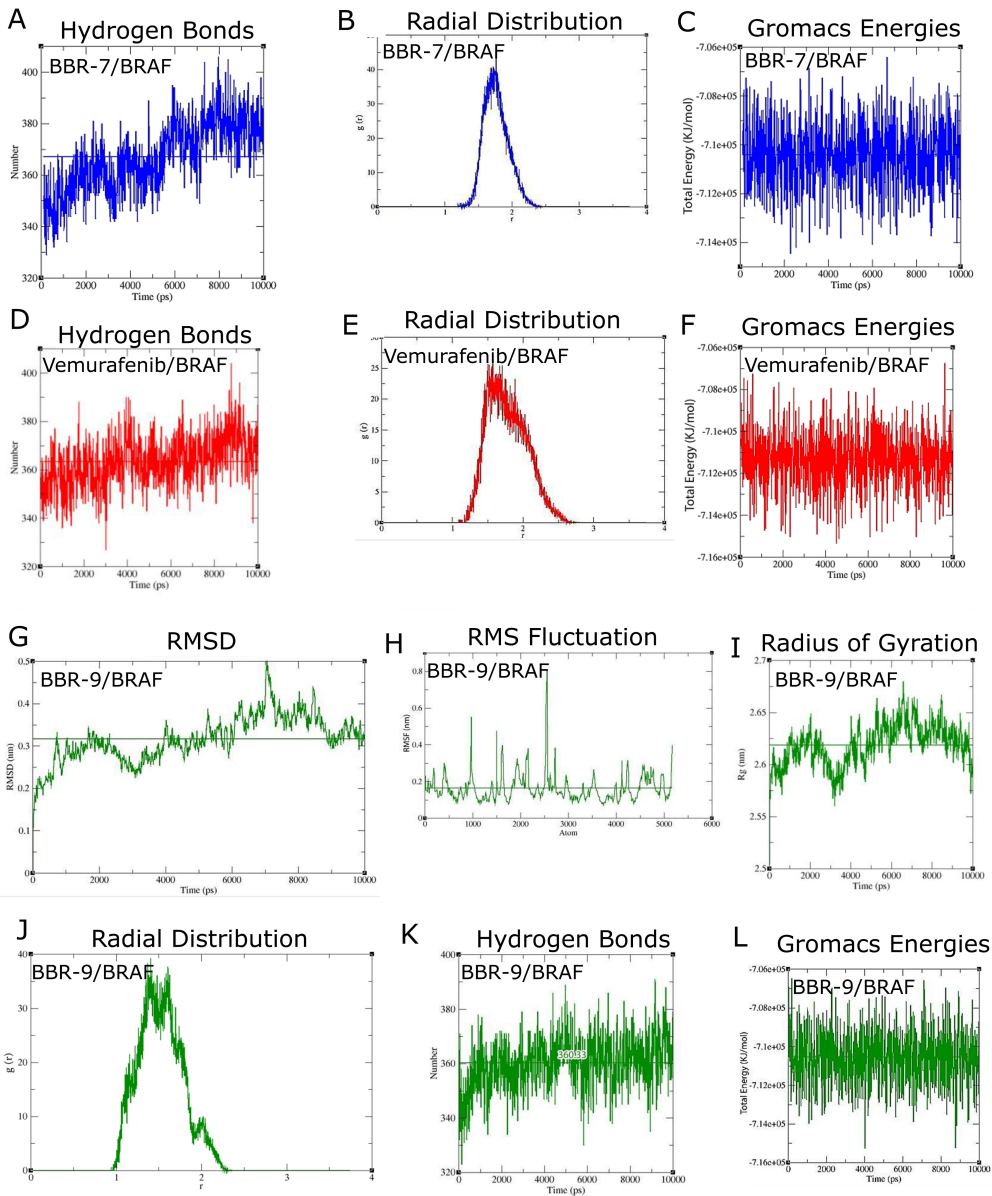
Molecular dynamics simulations of BBR-7, BBR-9, and vemurafenib. **(A-C)** BRAF/BBR-7; **(A)** The number of hydrogen bonds computed over time; **(B)** Chart of radial distribution function (RDF); **(C)** Total energies computed over time; **(D-F)** BRAF/Vemurafenib; **(D)** The number of hydrogen bonds computed over time; **(E)** Chart of RDF; **(F)** Total energies computed over time; **(G-L)** BRAF/BBR-9; **(G)** Root-Mean-Square Deviation (RMSD) chart; **(H)** Root-Mean-Square Fluctuation (RMSF) chart; **(I)** Average radius of gyration over time; **(J)** Chart of RDF; **(K)** The number of hydrogen bonds computed over time; **(L)** Total energies computed over time. Horizontal lines show the average of the parameter.

**Fig 5:**
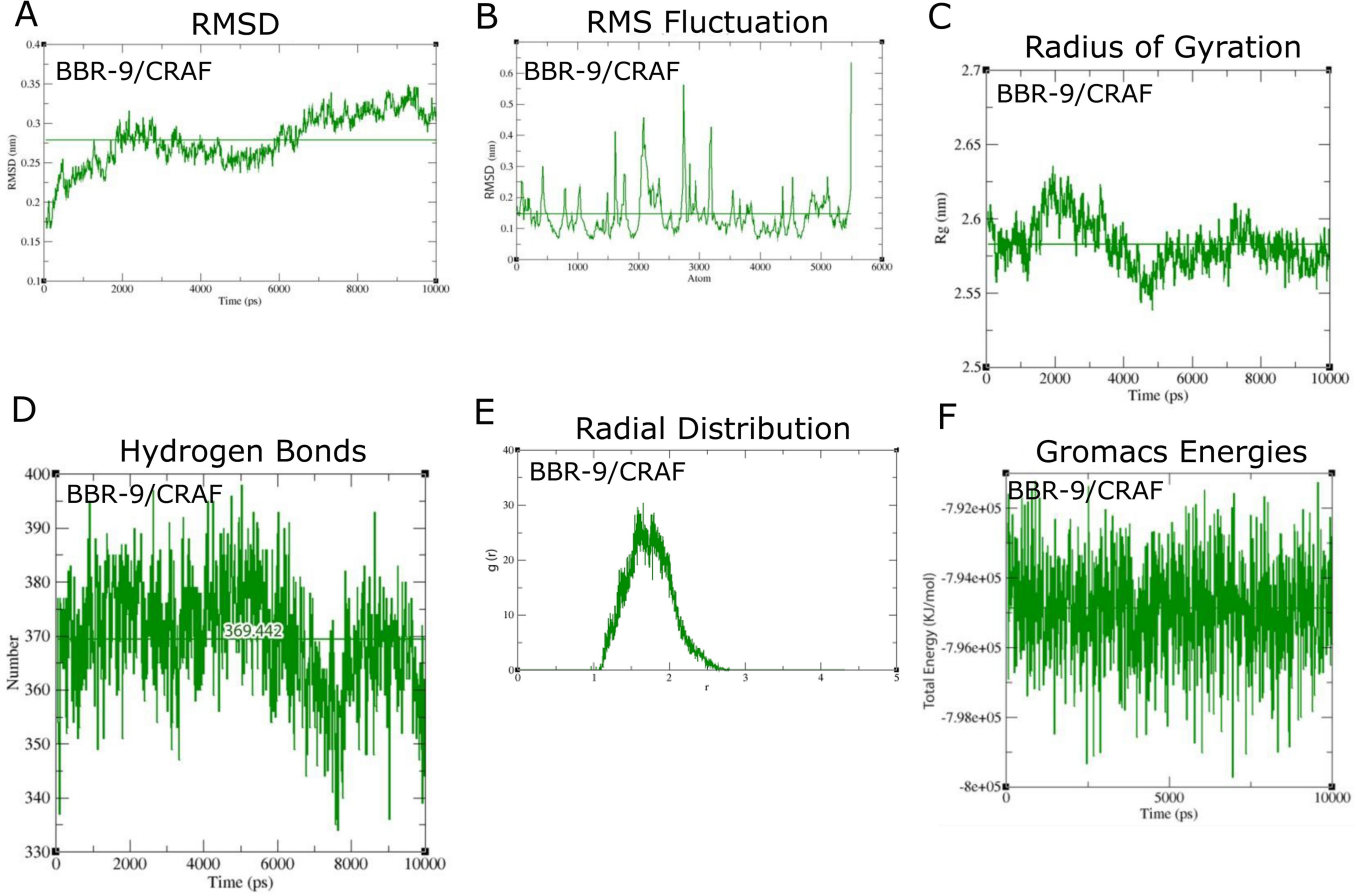
Molecular dynamics simulations of BBR-9 against CRAF. **(A)** RMSD chart; **(B)** RMSF chart; **(C)** Average radius of gyration over time; **(D)** The number of hydrogen bonds computed over time; **(E)** Chart of RDF; **(F)** Total energies computed over time. Horizontal lines on the chart show the average of the parameter.

By comparing the results of docking between BBR derivatives and several protein structures, we predicted that BBR-9 and BBR-7 would be BRAF inhibitors and BBR-9 would be a CRAF inhibitor. To show the stability and system energies of complexes, 10 ns MD simulations were conducted using Gromacs. RMSDs of around 0.3nm, with RMS fluctuations of less than 0.2nm, showed the stability of the interactions (**Figs 4 and 5**). During MD simulations, vemurafenib was used as a positive control. As the experimental property of vemurafenib was already reported, this comparison was used to predict our novel candidates, BBR-7 and BBR-9, as BRAF inhibitors. Modifications to protein motifs were evaluated using the radius of gyration (Rg). Rg provides information on total protein volume distribution in spherical states and indicates molecular shape over time. For BRAF/BBR-9 interaction **(Fig 4G–4L**), Rg was higher than CRAF/BBR-9 (**Fig 5**).

To validate the interactions between RAF kinases and BBR-9, radial distribution functions (RDF or g(r)) were computed. RDF demonstrates the distance between each molecule’s center of mass [36]. The g(r)s of BBR-9 and BBR-7 for BRAF were higher than that of BRAF bound to vemurafenib (**Fig 4**); however, the average distances (horizontal axis) between inhibitors and targets for all ligands were approximately 2Å, which shows the length of effective interactions. To confirm molecular docking, we computed the average number of H-bond and thermodynamic parameters of RAFs/ligand complexes (**Table 5**).

**Table 5:** Energies and the average number of H-bonds computed by Gromacs.

Molecular system	Parameter	Average	Err. Est.	RMSD	Tot-Drift	Unit
BRAF/BBR-9	Potential	-865261	52	1034.88	-372.464	kJ mol^-1^
	Total energy	-710511	53	1336.29	-372.717	kJ mol^-1^
	Enthalpy	-710471	53	1336.31	-372.707	kJ mol^-1^
	Temperature	299.995	0.0074	1.3278	-0.00049	K
	Pressure	0.971	0.48	106.163	0.121	Bar
	Hydrogen bonds	360.33	-	-	-	Number
CRAF/BBR-9	Potential	-969647	100	1115.47	-686.433	kJ mol^-1^
	Total Energy	-794859	100	1429.92	-692.926	kJ mol^-1^
	Enthalpy	-794815	100	1429.94	-692.927	kJ mol^-1^
	Temperature	299.997	0.0019	1.2438	-0.01115	K
	Pressure	1.589	0.35	98.1146	0.65583	Bar
	Hydrogen bonds	369.442	-	-	-	Number
BRAF/BBR-7	Potential	-865160	27	-nan	-155.213	kJ mol^-1^
	Total energy	-710393	27	-nan	-152.817	kJ mol^-1^
	Enthalpy	-710354	27	-nan	-152.8	kJ mol^-1^
	Temperature	299.983	0.0062	-nan	0.00464	K
	Pressure	1.47	0.18	-nan	0.2555	Bar
	Hydrogen bonds	367.12	-	-	-	Number
BRAF/Vemurafenib	Potential	-866099	38	1028.22	-257.798	kJ mol^-1^
	Total energy	-711311	39	1329.18	-257.934	kJ mol^-1^
	Enthalpy	-711271	39	1329.2	-257.925	kJ mol^-1^
	Temperature	299.997	0.0069	1.3267	-0.00028	K
	Pressure	0.85	0.35	105.381	0.2555	bar
	Hydrogen bonds	363.35	-	-	-	Number

### BRAF binding efficiency indices of BBR derivatives

Based on docking results, Binding Efficiency Indices (BEIs) were calculated to optimize the thermodynamics of docking based on the physicochemical properties of each ligand. BEIs are enzyme-specific indices for each molecule. To calculate efficiency indices, we combined molecular properties and docking information to provide quantitative parameters of the specificity of each compound for the BRAF kinase. Then, chemical and docking interactions of different ligands were compared, and bivariate plots were created to determine those that most potently antagonized BRAF. To calculate the difference of BEIs presented in **Table 6**, we used formulas and definitions described by Abed-Zapatero [37–40].

**Table 6:** Different ligand efficiency indices measured for different ligands docked to BRAF.

Ligand	N+O^1^	nha^2^	MW (kD)	LogP	TPSA (Å2)	pK_i_	NSEI^3^	NBEI^4^	nBEI^5^	mBEI^6^	LLE^7^
BBR	5	25	0.3364	2.473	39.93	5.90	1.22	0.24	7.48	5.62	3.61
BBR-2	6	30	0.4062	3.734	57.87	4.85	1.08	0.22	7.96	6.08	2.75
BBR-3	6	29	0.3922	3.165	57.87	5.35	0.98	0.20	7.36	5.47	2.74
BBR-4	5	24	0.3221	2.152	50.93	5.68	1.16	0.24	7.19	5.31	3.66
BBR-5	5	24	0.3221	2.152	50.93	5.78	1.22	0.26	7.49	5.61	3.96
BBR-6	6	27	0.3641	2.343	57.87	5.69	1.07	0.24	7.85	5.98	4.71
BBR-7	5	35	0.4812	3.65	40.16	4.53	1.32	0.19	8.15	6.28	2.96
BBR-8	5	26	0.3501	2.896	39.93	5.39	1.24	0.24	7.62	5.74	3.31
BBR-9	5	35	0.4791	4.5	40.16	4.87	1.36	0.20	8.35	6.48	2.31
BBR-10	5	36	0.4952	4.73	40.16	4.98	1.31	0.18	8.13	6.25	1.84
Vemurafenib	6	33	0.4891	2.786	96.01	6.26	1.04	0.19	7.78	5.92	3.47
Sorafenib	7	32	0.4641	1.568	91.82	6.21	0.89	0.19	7.72	5.90	4.64

^1^Number of polar atoms

^2^Heavy atoms (non-hydrogen atoms)

^3^Polarity-based binding efficiency index (NSEI = pK_i_/(N+O))

^4^Size-based binding efficiency (NBEI = K_i_/(nha))

^5^Atom-based logarithmic binding efficiency index (nBEI = pK_i_+Log [nha])

^6^MW-based logarithmic binding efficiency index (mBEI = pK_i_+Log [MW])

^7^Ligand lipophilicity efficiency (LLE = pK_i_-LogP).

Valuable chemical properties of drugs used in efficiency index calculations include molecular size, such as the number of atoms [non-hydrogen (nha) and electronegative] and lipophilicity or polarity (e.g., TPSA, LogP *etc*.) of ligands affecting drug absorbance and distribution. For better absorbance, the TPSA (topological polar surface area) should be lower than 100Å^2^. The MW-based logarithmic BEI (mBEI) and atom-based logarithmic binding efficiency index (nSEI) for BBR-9 were 6.48 and 8.35, respectively. Using different molecular properties (MW, TPSA, and the number of atoms), Abad-Zapatero reported different BEIs based on different logarithmic and nonlogarithmic numbers of atoms; however, nBEI, mBEI, and NSEI are three parameters supported by Abad-Zapatero, by which the best compounds against a certain target can be predicted. This novel prediction is a combination of chemical structure and thermodynamics (docking) results [41].

**Fig 6:**
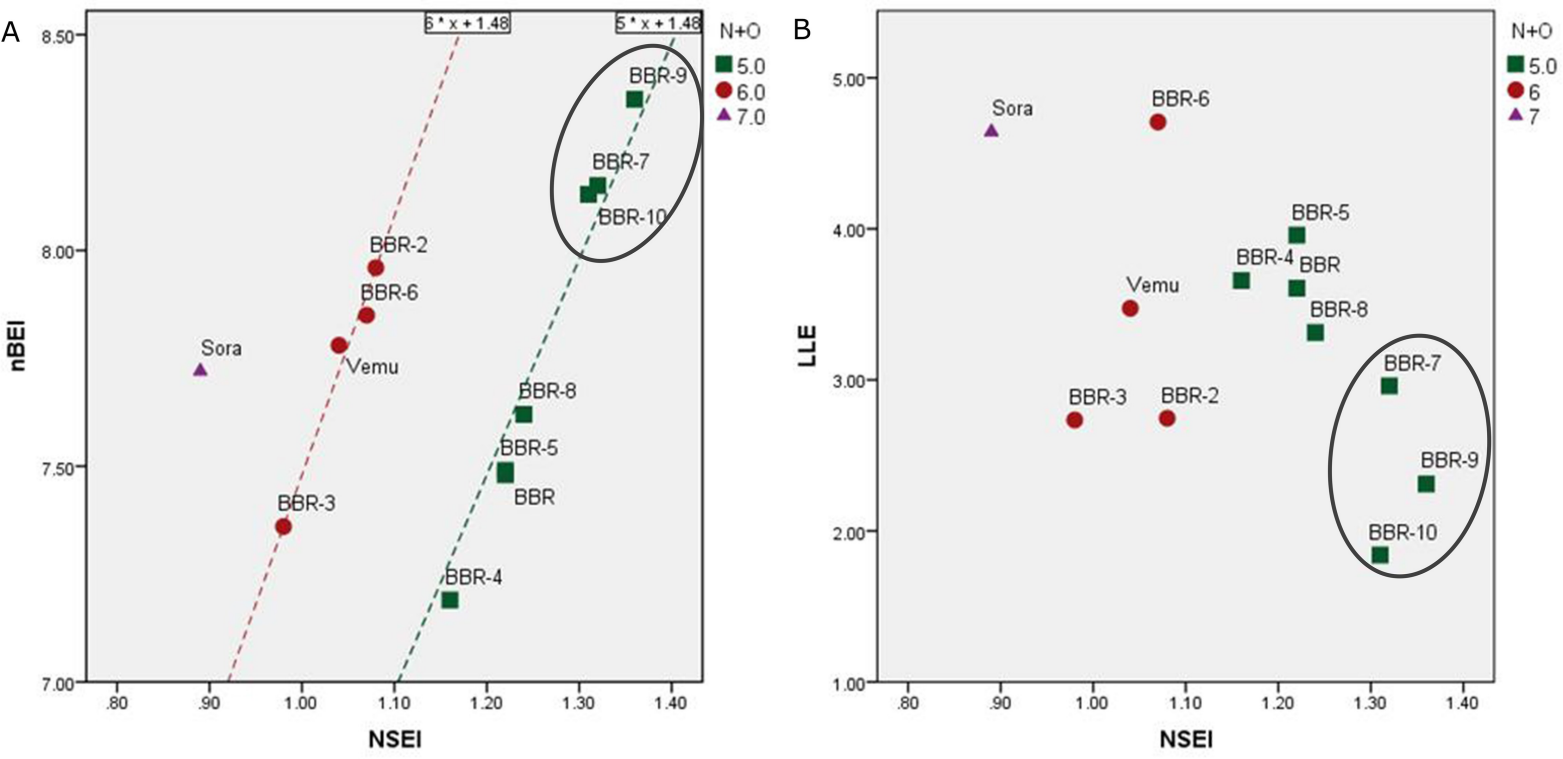
Plots showing the relationship of each ligand to its corresponding size- and polarity-based indices docked to BRAF. Horizontal axis is based on polarity and the vertical axis is size-dependent. There must be a balance between hydrophobicity and polarity of each ligand for suitable absorbance and distribution. **(A)** nBEI vs. NSEI. The formula of each regression line was predicted based on Y = NPOL.X+Log[nha] shown on the top of each line. The coefficient is the number of polar atoms (N+O), while the constant is the logarithm of the number of heavy atoms: nBEI = pKi+Log[nha]; binding efficiency index is based on the logarithmic number of heavy atoms; NSEI = pKi/(polar atoms); surface efficiency index is based on the integer number of polar atoms; **(B)** LLE vs. NSEI. LLE = pKi-LogP, lipophilicity index.

While we generated bivariate plots based on nBEI versus NSEI, we found that BBR-9, BBR-7, and BBR-10 were located in the top right corner of each plot, indicating they were the most potentially predicted BRAF antagonists (**Fig 6**). In contrast to vemurafenib and sorafenib, of which the LLE index is higher, BBR-9, BBR-7, and BBR-10 have much higher NSEI than LLE, meaning that the highest polarity of these ligands, especially BBR-9, may have a higher impact on the interactions between ligand and polar residues such as Lysine.

## Discussion

BRAF mutations have been detected in 7% of all cancers and in 66% of melanomas and 12% of colorectal cancers. As such, the FDA has approved BRAF inhibitors for cancer treatment. However, BRAF can alternatively activate CRAF leading to resistance to BRAF inhibitors. Here, for the first time, we analyzed anti-BRAF activity of BBR and demonstrated that it targets BRAF as a part of its anti-cancer activities.

RAF kinases are located upstream of the MAP kinase pathway and downstream of receptor tyrosine kinases (RTKs) (e.g., EGFR and Her2) and RAS. RTKs and RAS are mutated in 90% of pancreatic cancers, 30% of breast cancers, more than 50% of carcinomas, 30% of acute myelogenous leukemia, 30% of liver cancers, 55% of follicular thyroid cancers, 60% of undifferentiated papillary cancers, 35% of lung adenocarcinomas, and 10% of kidney cancers [13]. Potent RAF inhibitors can significantly block signals transduced by over-activated RTKs and RAS [42].

BBR attenuates the MAPK/ERK/RAF pathway, affecting cell proliferation in many types of cancer [43]. In addition, *in vitro* and *in silico* studies have shown that BBR reduces levels of ERK and p38 MAPK [44]; BBR also silences BRAF/ERK signaling in melanoma cells [8]. In the present study, we predicted that BRAF and CRAF were additional targets of BBR that affect ERK/p38 as downstream phosphate acceptors of BRAF, as BBR is bound to the position that ATP is normally bound to. Since BBR has cytotoxic effects, identifying new BRAF inhibitors with less cytotoxicity is of great importance to cancer treatment [1].

Position 13 of BBR plays a major role in reducing MAPK pathway activity. HWY 289 and HWY 336, two BBR derivatives with added aromatic branches on position 13, suppress cell proliferation in fission yeast (*Schizosaccharomyces pombe*) by inhibiting the MAPK cascade. The minimal inhibitory concentration (MIC) of HWY 289 and HWY 336 are 29.52μM and 11.83μM, respectively [35]. In contrast to BBR which does not completely inhibit proliferation of wild type *S*. *pombe*, HWY 289 and HWY 336 completely block the proliferation of this yeast [35]. After docking hundreds of BBR derivatives obtained from the PubChem database, we generated BBR-10 with the addition of [4-(trifluoromethyl) methyl benzene] to position 13. After docking BBR-10 against BRAF crystals, it was bound to the BRAF active site with an average Ki of 0.269μM, while the Ki of BBR was 0.832μM, three times higher than that of BBR-10. These results identified BBR-10 as a BBR-derived lead candidate for BRAF inhibition.

The structure-activity relationship (SAR) showed that replacing the methoxyl group at position 9 with an ester moiety could significantly increase the anti-tumor activity of BBR [45]. In addition to BBR-10, we added a trifluorobenzel group to methoxy group located at the positions 9 (BBR-9) and 10 (BBR-7). The trifluorobenzel structure is similar to the active part of sorafenib. Docking results of BBR-7 and BBR-9 against BRAF and CRAF kinases showed that they are both more effective than BBR-10, as BBR-9 was bound to BRAF and CRAF with Ki values of 0.155μM and 0.265μM, respectively. Our results also showed that BBR-9 might be effective as a dual RAF inhibitor. The direct effects of BBR derivatives against BRAF and CRAF kinases had not yet been reported, but, here, for the first time, we report three cycloprotoberberines as lead compounds against RAF kinases.

## Conclusion

In the present study, we sought to identify drug candidates that simultaneously inhibit BRAF and CRAF kinases. We predicted that BBR-9 would be a strong dual RAF inhibitor. In addition to BBR-9, BBR-7 and BBR-10 were also identified as attractive candidates against RAF kinases. *In vitro* and *in vivo* evaluation of these inhibitors may establish them as lead compounds to treat cancer. In addition to direct RAF inhibitory of BBR derivatives, these compounds may act as indirect inhibitors of over-activated RTKs and RAS for different types of cancer.
